# Design of debunking the frailty–sarcopenia–ADT axis in metastatic prostate cancer with multicomponent exercise: the FIERCE trial protocol

**DOI:** 10.3389/fspor.2025.1602123

**Published:** 2025-10-23

**Authors:** Rebekah L. Wilson, Anderson Vulczak, Alicia K. Morgans, Mary Norris, Jocelyn Greer, Jennie Votta, Maria Vamvini, David J. Einstein, Hajime Uno, Michael Rosenthal, Matthew G. Vander Heiden, Christina M. Dieli-Conwright

**Affiliations:** ^1^ Department of Medical Oncology, Dana-Farber Cancer Institute, Boston, MA, United States; ^2^ Department of Medicine, Harvard Medical School, Boston, MA, United States; ^3^ Department of Biomolecular Sciences, School of Pharmaceutical Sciences of Ribeirão Preto, University of São Paulo, São Paulo, Brazil; ^4^ Joslin Diabetes Center, Boston, MA, United States; ^5^ Genitourinary Oncology Program, Beth Israel Deaconess Medical Center, Boston, MA, United States; ^6^ Department of Imaging, Dana-Farber Cancer Institute, Boston, MA, United States; ^7^ Department of Radiology, Brigham and Women’s Hospital, Boston, MA, United States; ^8^ Department of Biology, Koch Institute for Integrative Cancer Research, Massachusetts Institute of Technology, Cambridge, MA, United States

**Keywords:** prostate cancer, exercise, frailty, sarcopenia, androgen deprivation therapy

## Abstract

**Introduction:**

The incidence of metastatic prostate cancer (mPCa) is increasing despite a decrease in the prevalence of prostate cancer (PCa). Androgen deprivation therapy (ADT), the mainstay of systemic treatment for mPCa, is associated with numerous side effects, including a decline in muscle mass and physical function, which lead to the exacerbation of age-related frailty and sarcopenia. Exercise plays a key role in ameliorating or preventing the progression of ADT-related side effects and in improving muscle mass, fitness, and strength. However, exercise interventions in patients with mPCa have been understudied, with a lack of studies focusing on frailty and sarcopenia and the mechanisms by which exercise could address these issues.

**Purpose:**

Thus, we have designed the FIERCE trial to assess the effects of a 16-week multicomponent exercise intervention, encompassing resistance and aerobic training, on frailty and sarcopenic status and their potential mechanistic biomarkers, as well as on cancer cell proliferation (NCT06040125).

**Methods:**

The FIERCE trial is a prospective study aiming to recruit 80 pre-frail/frail men with mPCa receiving ADT who will be randomized to either an exercise or an attention control group. The 16-week exercise intervention will include thrice-weekly, clinic-supervised, resistance exercise circuit training and self-directed home-based aerobic exercise. The attention control group will receive a stretching program and will be offered the exercise program following the study period. The primary outcome is frailty, measured by the Fried frailty phenotype (i.e., muscle loss, exhaustion, physical activity, gait speed, and strength) and frailty-associated biomarkers [IL-6, TNF-α, C-reactive protein (CRP)]. Secondary outcomes include sarcopenia, measured using dual-energy x-ray absorptiometry scans and sarcopenia-associated muscle biopsy-driven biomarkers (myokines and insulin pathway markers). An exploratory outcome will assess how exercising patient-derived plasma will affect the proliferation of prostate cancer cells (LNCaP cells).

**Conclusion:**

This first-of-its-kind study targets a vulnerable, understudied population: frail men with mPCa. If successful, our findings will establish the efficacy of a multicomponent exercise intervention on frailty and sarcopenic status, providing the foundation for future larger phase II and III trials to confirm the findings and potentially establish exercise as a safe and necessary part of the standard of care for frail metastatic prostate cancer patients.

## 1 Introduction

Androgen deprivation therapy (ADT), the mainstay systemic treatment for men with recurrent and/or metastatic prostate cancer (mPCa), works by depriving the body of hormones such as testosterone. Given the critical role testosterone plays in activating lipolysis and promoting lean mass hypertrophy, substantial body composition changes and loss of muscle strength and physical function can occur leading to the development or exacerbation of geriatric syndromes such as frailty and sarcopenia (1). Frailty, a comprehensive loss of functional reserve in multiple physiological systems, is a common clinical concern found in 40%–90% of men with PCa on ADT and is associated with a worse prognosis (2–5). Specifically, severely frail men showed an 86% higher risk of all-cause mortality and a 44% increased risk of cancer-specific mortality compared with their non-frail counterparts (4). Similarly, sarcopenia, characterized by a decrease in muscle mass, strength, and function, occurs in >80% of men with mPCa on ADT (6–8) and is an independent risk factor of cancer-specific mortality (9). An intervention that can address frailty and sarcopenia, alongside persisting ADT-related concerns, is critical to improve the clinical care of this population.

Aerobic and resistance training is a recommended complementary therapy for PCa patients and has been shown to prevent further deterioration of ADT-related adverse effects, such as skeletal muscle mass loss, fat mass gain, and reduced cardiorespiratory fitness and muscular strength (10–13). In addition, the inclusion of functional movements (e.g., balance) can maximize the beneficial effects of exercise on physical function, muscular strength, muscular endurance, and body composition (14–19). Within the context of frailty and sarcopenia, a multicomponent exercise intervention is recommended, given the multifaceted exercise stimulus that is required of an intervention to target the various characteristics of both syndromes (20). Nevertheless, men with mPCa are largely underrepresented in previous exercise oncology research, and no studies have specifically focused on frailty and sarcopenia in this population (20–23). Thus, a rigorously designed randomized controlled trial is warranted, which examines an exercise prescription specifically targeting the frailty–sarcopenia–ADT axis in frail men with mPCa.

Understanding whether exercise prevents or reverses frailty and sarcopenia, as well as the mechanisms implicated, is critical for improving health and quality of life in men with mPCa on ADT, yet the mechanisms by which exercise improves frailty and sarcopenia, particularly in the context of ADT, are unknown (7). Proposed mechanisms are improvements in systemic inflammatory and skeletal muscle tissue-related outcomes (7). Thus, we will investigate systemic inflammatory biomarkers associated with frailty [IL-6, TNF-α, C-reactive protein (CRP)] and muscle tissue-related outcomes associated with sarcopenia (myokine release and activation of insulin pathway-related biomarkers) to aid in refining explanations surrounding the frailty–sarcopenia–ADT axis with exercise (5, 24–29). In addition, exploring whether the effects of exercise are associated with biochemical cancer progression will provide insight into understanding the roles of exercise in long-term oncologic outcomes in the context of mPCa.

The overall objective of the FIERCE trial is to evaluate the efficacy of a 16-week multicomponent exercise intervention encompassing supervised, clinic-based circuit training utilizing resistance exercises and self-directed aerobic exercise on frailty and sarcopenia with an exploration of novel biomarkers at the intersection of frailty, sarcopenia, and disease progression. The primary outcome of this study is change in frailty score, measured by the Fried frailty phenotype (i.e., muscle loss, exhaustion, physical activity, gait speed, and strength) and frailty-associated biomarkers (IL-6, TNF-α, CRP). Secondary outcomes include change in sarcopenia status, measured using a computerized tomography (CT) scan and sarcopenia-associated muscle biopsy-driven biomarkers (myokines and insulin pathway markers). An exploratory outcome will assess how plasma from patients undergoing the exercise intervention affects prostate cancer cell proliferation (LNCaP cells). We hypothesize that the exercise group, compared with the attention control group, will exhibit the following: (1) a lower proportion of frail and pre-frail scores and improved individual scores of each frailty component; (2) improvements in systemic inflammatory biomarkers; (3) improved sarcopenia status; (4) increased gene and protein expression of muscle biopsy-driven anti-inflammatory myokines and insulin pathways; and (5) suppressed biochemical progression of PCa.

## 2 Methods

### 2.1 Study design

**Figure 1:**
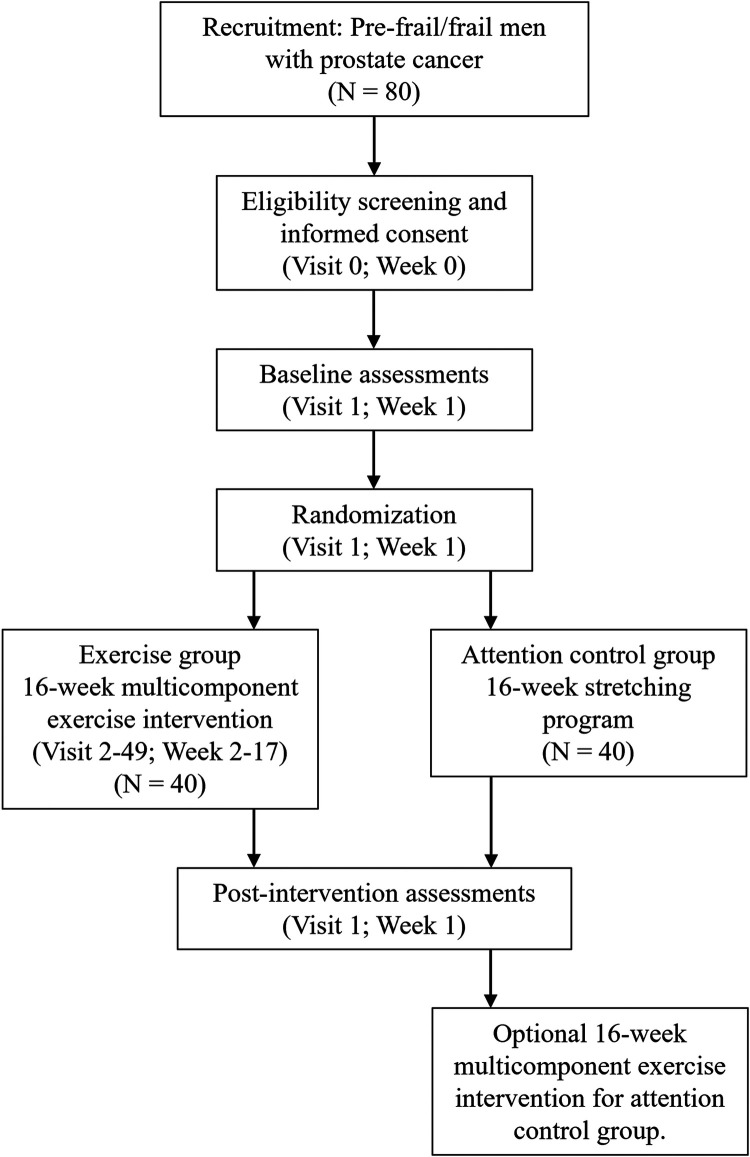
Study schema for the FIERCE trial.

The FIERCE trial is a two-arm randomized controlled trial underway at the Dana-Farber Cancer Institute (NCT06040125). Outcomes are measured at baseline (week 1) and at post-intervention (week 18). A total of 80 men diagnosed with metastatic prostate cancer, who have a history of or are currently receiving, androgen deprivation therapy and are considered pre-frail or frail, are randomized to one of two groups: (1) intervention group, who will receive a 16-week supervised, clinic-based, multicomponent exercise intervention in conjunction with self-directed aerobic exercise or (2) attention control group, who will receive a home-based stretching program. Those randomized to the attention control group will be offered the exercise program following the initial 16-week study period (Figure 1).

### 2.2 Participant eligibility

Men are eligible if they meet the following criteria: (1) diagnosed with metastatic prostate cancer; (2) aged >18 years; (3) have received at least 3 months of androgen deprivation therapy or are currently receiving at least 1 month of androgen deprivation therapy and are expected to remain on therapy for the duration of the study; (4) classified as pre-frail or frail as determined by the FRAIL scale (score of 1–2 = pre-frail; 3–5 = frail) (30–33); (5) medically cleared by their clinicians for exercise; (6) English-speaking; and (7) inactive (defined as engaging <60 min of moderate-to-vigorous aerobic exercise per week in the past month or <2 resistance exercise sessions per week in the past 4 months). Exclusion criteria include the following: (1) actively receiving chemotherapy or radiotherapy; (2) having unstable bone lesions; (3) having any uncontrolled comorbidity, condition, or contraindication that may prevent participation in exercise; and (4) receiving treatment for other active malignancy (except basal cell carcinoma).

### 2.3 Recruitment and informed consent

Participants are currently being recruited through clinician referral and screening of participant lists at the Dana-Farber Cancer Institute, Boston, MA, USA, as well as by way of advertisements in participant waiting rooms, the Partners Rally Platform, and other relevant prostate cancer community groups and events. All participants require clinician clearance before being formally screened via phone. Phone screening comprises three short questionnaires to determine eligibility: the Godin Leisure Time Questionnaire (34) to assist in determining current exercise levels, the Physical Activity Readiness Questionnaire (35) to confirm health status and how this relates to undertaking an exercise program, and the FRAIL questionnaire (30–33) to assess frailty status. Upon confirmation of eligibility and interest, participants consent via wet or electronic signature.

### 2.4 Ethics approval

The study has been approved by the Institutional Review Board at the Dana-Farber Cancer Institute (IRB #23-109). The studies were conducted in accordance with the local legislation and institutional requirements. The participants provided their written informed consent to participate in this study.

### 2.5 Randomization and blinding

After baseline testing, participants are randomly allocated to either the exercise or attention control group using a 1:1 ratio and a permuted blocked design with varying block sizes and stratified by frailty status as assessed by the FRAIL scale. The study biostatistician and co-investigator (HU) prepared the randomization list before study start-up, after which the investigators, who are blinded to this list creation, access it through a web-based application to conduct randomization and subsequently verbally inform the participants of their group allocation. Testers and participants are blinded to group allocation during baseline testing. Owing to the nature of the intervention and logistical limitations, participants and study staff cannot be blinded to the intervention allocation.

### 2.6 Measurements

#### 2.6.1 Overview

Testing is completed over 2 days at baseline and post-intervention. Order of days is divided by a collection of biospecimens (i.e., blood and muscle tissue) and non-physical tests (i.e., questionnaires and body composition) completed on 1 day, and all other physically demanding tests are completed on the other (∼2–3 days apart). This separation of measures is to ensure participants can recover from the biopsy and not complete any physically demanding activities that may influence blood or muscle biomarkers. All measures are collected at all time points by study staff members trained in exercise oncology unless otherwise specified (Table 1).

**Table 1:** Outcome measures by study visits for the FIERCE trial.

Timepoint	Week 0	Week 1–17	Week 18	Week 19–34^a^
Enrollment				
Eligibility screen	X			
Phone screen	X			
Informed consent	X			
Randomization	X			
Interventions				
Circuit training + self-directed aerobic exercise		X		X (optional)
Attention control		X		
Assessments				
Fried frailty phenotype measures	X		X	
Muscle biopsy (optional)	X		X	
Blood biomarkers	X		X	
Anthropometrics	X		X	
Patient-reported outcomes	X		X	
Cardiorespiratory fitness	X		X	
Physical function	X		X	
Strength	X		X	
Physical activity	X	X	X	
Diet	X		X	
Exit survey			X	

^a^ Applies to the attention control group and is optional.

#### 2.6.2 Primary outcomes

##### 2.6.2.1 Fried frailty phenotype

The Fried frailty phenotype is a composite score of the five measures: muscle loss, exhaustion, physical activity, slowness, and weakness. Participants receive a score of 1 (experience muscle loss, exhaustion, etc.) or 0 (do not experience) for each characteristic, with a total score of 1–2 identified as pre-frail and ≥3 as frail (36, 37). The Fried frailty phenotype was selected as it offers the most accurate way to capture frailty status using objective measures with pre-determined cutoff points (1, 36). A modified version of the Fried frailty phenotype was selected to tailor to the known effects of ADT, e.g., muscle loss, to ensure frailty was captured in a way that was tailored to the studied population, as has been proposed by other prostate cancer studies assessing frailty (1, 36).

###### 2.6.2.1.1 Muscle loss

A skeletal muscle index (SMI—calculated as appendicular skeletal muscle mass / height^2^) sarcopenia score of ≤7.26 kg/m^2^, as assessed by dual-energy x-ray absorptiometry (DXA) scan, is the cut point used for identifying muscle loss = 1 point (6, 38). In addition, in a subset of patients with available standard of care computed tomography (CT) scans within ∼1 month of baseline and post-intervention time points, sarcopenia will also be assessed with a skeletal muscle index of ≤52.4 cm^2^/m^2^ as the cut point used for identifying muscle loss = 1 point (39). Skeletal muscle area is measured on axial CT images at the level of the L3 vertebral body using a validated, fully automated algorithm (40). SMI is calculated as the ratio of muscle area (cm^2^) to squared height (m^2^). A CT series will be analyzed into two steps: (1) a convolutional neural network model is used to identify a slice at the L3 level, and (2) the chosen slice is passed to a segmentation model to estimate the cross-sectional areas of muscle (40).

###### 2.6.2.1.2 Exhaustion

Exhaustion is assessed using the vitality score from the Short Form Health Survey (SF-36), a 36-item questionnaire assessing quality of life. A cut point of <50 for those 50–65 years and <40 for those >65 years is used to identify exhaustion = 1 point (36, 37, 41).

###### 2.6.2.1.3 Low physical activity

The Community Health Activities Model Program for Seniors’ physical activity questionnaire is used to assess physical activity, with low activity defined as <383 kcals = 1 point (36, 37). This is a 41-item questionnaire asking about time spent undertaking certain physical activities (42).

###### 2.6.2.1.4 Slowness

Usual gait speed is assessed over a 15-foot flat surface distance with an average time over two attempts of ≥7 s for participants ≥173 cm tall or ≥6 s for those <173 cm tall considered as “slow” = 1 point (36, 37). In addition, the participant is asked to walk at their “fast” pace for the 15-foot distance a further two times, but this is not utilized for the Fried frailty phenotype composite score.

###### 2.6.2.1.5 Weakness

The chair stand test is used to assess leg strength, where a time ≥12 s to complete five chair stands is considered weaker = 1 point (36, 37). Participants are asked to sit in a chair with their hands crossed over their chest. While keeping the feet flat and back straight, participants rise to a full standing position and then sit back down, repeating five stands as fast as they can.

##### 2.6.2.2 Blood biomarkers

A trained phlebotomist performs a fasting blood draw, obtaining both serum-separating tubes and ethylenediaminetetraacetic acid (EDTA) samples. After being processed and aliquoted, the blood serum and plasma samples are stored in a −80°C freezer for later batched analysis. Analysis of blood through commercially available kits (Thermo Fisher Scientific) will be used for biomarkers relating to inflammation, frailty, or sarcopenia (i.e., IL-6, TNF-α). Plasma will also be used for exploratory studies assessing effects on prostate cancer cell proliferation.

#### 2.6.3 Secondary outcomes

##### 2.6.3.1 Sarcopenia status

As described under assessing muscle loss as part of the Fried frailty phenotype.

##### 2.6.3.2 Muscle biopsy

Skeletal muscle biopsies of the vastus lateralis are optional, where participants are asked at the time of consent if they wish to partake. Skeletal muscle-derived biomarkers to be assessed include myokines [IL-6, IL-15, secreted protein acidic and rich in cysteine (SPARC), oncostatin M (OSM), decorin, irisin] and insulin pathway-related biomarkers (GLUT-4 receptors, IGF-1, IGFBP-3). The muscle biopsy is performed by a qualified nurse practitioner and/or physician assistant.

The local area (quadriceps thigh muscle) is anesthetized with a numbing spray, followed by an injection of lidocaine. A cannula (∼13.5-gauge) is then inserted into the vastus lateralis muscle perpendicularly, such that it pierces through the layer of fascia surrounding the muscle fibers. To obtain the biopsy, the needle is fed through the cannula, and the biopsy trigger is activated, resulting in a spring-loaded, rapid collection of ∼10–15 mg muscle tissue. The cannula remains inserted into the muscle, the needle is removed, and the procedure is repeated until an adequate sample is collected. After sampling is complete, the cannula is removed, and pressure is applied to the area to stop any potential bleeding, with a bandage placed over the biopsy site to promote healthy healing. The muscle sample is briefly washed on clean gauze and frozen using liquid nitrogen for storage at −80°C for later batched analysis. The biopsies occur following a 12 h fast. The post-intervention biopsy is performed at a distance of ∼1 cm from the first incision and 72–96 h after the last training session for participants in the exercise to reflect resting levels of mRNA expression (43).

#### 2.6.4 Exploratory outcomes

##### 2.6.4.1 Prostate cancer cell proliferation

The proliferation rate of LNCaP cells is assessed using plasma samples at baseline and at post-intervention. LNCaP cell line is grown in ATCC-formulated RPMI 1640 media supplemented with 5% fetal calf serum (FCS) and 1% penicillin–streptomycin (ATCC Manassas, VA, USA). LNCaP cells (100 µL) are seeded at a concentration of 50,000 mL in a 96-well plate containing either 5% FCS with or without the addition of 5% human plasma from test subjects in triplicate for 48 h to determine cell proliferation. As an alternative, patient serum collected could be used in lieu of FCS, and this will be experimentally determined. After plating the same number of cells for a defined period of time, final cell numbers will be determined by removing supernatant and fixing cells with 100 µL 4% paraformaldehyde in the plate for 20 min. Fixed cells are then incubated for an additional 20 min with 100 µL 2% crystal violet (Thermo Fisher Scientific, Edmonton, Canada) dye solution (0.1%, w/v, with ethanol 2%, v/v in 0.5 M Tris-C1, pH 7.80) (44). The stained cells are washed in tap water and solubilized with an SDS solution (0.1%, w/v, with ethanol 50%, v/v, in 0.5 M Tris-C1, pH 7.8; 100 µL per well) for 30 min. The crystal violet dye is released by the fixed cells into the supernatant, and the absorbance is measured by a Molecular Devices spectrophotometer (San Jose, CA, USA) at 600 nm.

#### 2.6.5 Covariates

##### 2.6.5.1 Participant characteristics, health behaviors, and medical history

Participant demographic and medical history data were collected at baseline from medical records, and participants completed questionnaires. Information collected includes age, gender, race, ethnicity, non-cancer medical history, cancer history, family cancer history, anthropometric history, smoking habits, alcohol intake, medication use, and vitamin and supplement use.

##### 2.6.5.2 Anthropometrics

Body composition is assessed via bioelectrical impedance analysis (BIA) using a validated device (Tanita 780, Arlington Heights, IL, USA). The device estimates body fat using an algorithm based on the user’s age, sex, height, and body weight. Waist and hip circumference is also obtained using a constant-tension tape measure.

##### 2.6.5.3 Participant-reported outcomes

Health-related quality of life is assessed using the European Organization for Research and Treatment of Cancer Quality of Life Questionnaire-C30 (45). A higher functional score represents a healthier level of functioning, a higher global health status represents a higher quality of life, and a higher symptom scale represents a higher level of symptoms. Sleep quality is assessed using the Pittsburgh Sleep Quality Index (PSQI), which contains 19 questions evaluating seven domains of sleep—subjective sleep quality, sleep latency, sleep duration, habitual sleep efficiency, sleep disturbances, use of sleep medication, and daytime dysfunction (46, 47). The Functional Assessment of Cancer Therapy—Bone Pain (FACT-BP) is used to assess cancer-related bone pain and its effect on quality of life (48). The Brief Pain Inventory (BPI) is used to assess the impact of general pain on quality of life. The BPI includes a sensory and reactive dimension and has been previously validated in breast cancer survivors (49). The Exercise Benefits/Barriers Scale (EBBS) is used to assess barriers to exercise (50). Finally, the expanded Prostate Cancer Index Composite-26 is used to measure health-related quality of life across five prostate cancer-specific domains (51).

##### 2.6.5.4 Cardiorespiratory fitness

To assess maximal oxygen consumption (VO_2peak_) and accurately prescribe the intensity for the self-directed aerobic exercise, a maximal cardiopulmonary exercise test is completed on a cycle ergometer (ErgoSelect 100; Ergoline) using an incremental ramp protocol (52). Participants complete a 3 min warm-up with no resistance and then start the ramp protocol at 10 W and proceed into an incremental ramp protocol increasing 10 W every minute until volitional fatigue. Cadence is kept between 60 and 70 revolutions per minute. Heart rate (HR; FT4; Polar, USA) and rate of perceived exertion (Borg scale 1–10) are recorded every minute and at the end of the test. Expired gas analysis (TrueOne 2400; ParvoMedics, Inc.) is used to measure VO_2peak_. The results of this test are used to calculate the target heart rate for the self-directed aerobic exercise.

##### 2.6.5.5 Physical function

Functional power is measured using a stair climb test that has been successfully performed and correlated with lower-extremity power and mobility performance in older adults (53). Participants are instructed to ascend a flight of 10 stairs one step at a time as quickly as possible. Timing begins when one foot steps on the third stair and ends when one foot reaches the ninth stair. Time is recorded to the nearest 0.01 s, where three trials are completed, and the fastest is used to calculate power (54).

Mobility is assessed using the Timed Up and Go (TUG) test, which has been shown to predict immediate fall risk better than static balance tests or isometric muscle strength (55). Participants begin seated in a chair, walk to a line on the floor 3 m from the chair, turn around, and return to the same seated position as quickly and safely as possible. Scores are taken as the time to complete the task once, where an average of the time for three trials is calculated.

##### 2.6.5.6 Muscular strength

Grip strength is measured using a handheld dynamometer on the participant's dominant hand. The subject is asked to grip the handle of the dynamometer with one hand using as much grip pressure as possible while holding for 3–5 s. The average of three attempts is calculated.

One repetition maximum (1 RM) is estimated from 10 RM muscle strength tests on two exercises: (1) leg press and (2) chest press. One RM values are calculated and reported using validated equations (56, 57). Following a warm-up, a maximum of five attempts is given to reach the final 10 RM load with a 1–2 min rest period between attempts.

##### 2.6.5.7 Physical activity

Participants are provided an accelerometer (ActiGraph, Pensacola, FL, USA) to wear for 7 days at all times except for when swimming, bathing, and/or sleeping. The device records activity data and is electronically transferred to a computer via USB cable at the time of completion. In addition, control participants are also given weekly physical activity logs to complete over the initial 16-week intervention period.

##### 2.6.5.8 Diet

An automated self-administered 24 h dietary assessment tool is used to assess recent dietary patterns for two weekdays and one weekend day (58). This is completed via an online portal over 3 non-consecutive days where possible.

##### 2.6.5.9 Exit survey

A study-tailored questionnaire evaluating intervention satisfaction is provided at post-intervention testing only. This survey includes questions related to the following: (1) timing of the intervention; (2) length of sessions; (3) telephone-based approach; (4) usefulness of the session content in terms of physical activity; and (5) satisfaction with interventionists. A separate exit survey is provided to the two groups and tailored to the respective exercise or stretching intervention completed.

### 2.7 Intervention

#### 2.7.1 Exercise group

Participants randomized to this group partake in a 16-week multicomponent exercise intervention consisting of a functional exercise warm-up and a resistance exercise circuit that is supervised in a clinic-based environment 3 days per week. The supervised sessions will be one-on-one, led by a certified exercise trainer in a hospital-based gym. Participants are also encouraged to complete self-directed home-based aerobic exercise on 4 days per week. Before beginning the supervised exercise, participants undergo a screening procedure to assess their bone pain level on a scale of 0–10. In the event bone pain is described, adjustments are made to the resistance exercise for that day to ensure the participant exercises in a pain-free motion.

Clinic-based component: Sessions begin with a dynamic warm-up of functional exercises that target aspects of balance, coordination, agility, and functional movements associated with tasks of everyday living (example exercises include calf raises, arm swings, and wrist rotations). The warm-up is followed by a circuit-based resistance program that is performed by alternating upper and lower body exercises in a single circuit. Participants complete four cycles (one cycle = 4 weeks) over the 16-week intervention. An orientation session (counting as their first session) takes place to introduce the study and exercises to the participant and to walk them through how a session will be run and what to expect in each cycle. The first session of each new cycle involves RM testing of each exercise to be prescribed in that 4-week cycle. During any session that is not considered a de-loading session, a 5%–10% increase in weight may occur when the participant can do >3 repetitions with good technique beyond what is prescribed and reports a low rate of perceived exertion (i.e., <7); this ensures participants are consistently working at the assigned RM. In each 4-week cycle, new exercises (Table 2) are introduced to provide variety and alternative stimuli to the body, while targeting the same muscle groups each session/cycle. While in Table 2, the primary exercises are described, alternative exercises that target the same muscle group may be used, subject to participant health and abilities. With this variety in exercises also comes variety in equipment, where machine weights, dumbbells, body weight, and resistance band exercises are incorporated throughout the 16-week intervention. The exercise intervention is periodized to ensure progressive overload and training adaptations. The prescription will be at an intensity ranging from 8 to 15 RM, completing 2–3 circuits of 6–15 repetitions of each exercise (Table 3). In addition, within each week, there will be two higher intensity days and one de-load day, again to provide variety and prevent overload. Each session will conclude with a cooldown incorporating stretches.

**Table 2:** Example resistance exercises prescribed in each exercise cycle.

Exercise examples
Cycle 1: Machine-based Leg press, chest press, leg curl, seated row, leg extension, shoulder press
Cycle 2: Combination of machine and free weights Leg press, dumbbell bench press, dumbbell reverse lunges, seated row, dumbbell chair squat, dumbbell lateral raise
Cycle 3: Predominantly free weight-based Leg press, dumbbell bench press, resistance band deadlift, dumbbell bent over row, dumbbell chair squat, dumbbell shoulder press
Cycle 4: Combination of machine and free weights Leg press, chest press, glute bridge, alternating arm dumbbell row, dumbbell lunges, dumbbell shoulder press

**Table 3:** Intervention periodization for the FIERCE 16-week intervention.

Weekly session number	Resistance training (supervised in-person)			Aerobic training (self-directed at home)	
	Weight intensity	Circuits	Reps	Intensity	Time
Cycle 1 (Week 1–4)					
1	15 RM	2–3^a^	15	60%–65% HRR	15 min/day
2	15 RM	2–3^a^	12		
3	15 RM	2–3^a^	15		
Cycle 2 (Week 5–8)					
1	12 RM	3	12	65%–70% HRR	20 min/day
2	12 RM	3	10		
3	12 RM	3	12		
Cycle 3 (Week 9–12)					
1	10 RM	3	10	70%–75% HRR	30 min/day
2	10 RM	3	8		
3	10 RM	3	10		
Cycle 4 (Week 13–16)					
1	8 RM	3	8	75%–80% HRR	30 min/day
2	8 RM	3	6		
3	8 RM	3	8		

HRR, heart rate reserve.

^a^ Two circuits are completed in the first week only.

Home-based component: Participants are offered an optional stationary bike to help complete self-directed aerobic exercise at home; however, participants may choose any form of aerobic exercise that suits their lifestyle. In addition, if they do not have one, participants are offered an activity monitor to monitor heart rate intensity. Aerobic exercise is completed on any day of the participant's choosing, 4 days per week, at an intensity of 60%–80% heart rate reserve for 15–30 min per day (Table 3). During the supervised sessions, exercise trainers record the reported home-based aerobic activity completed since the last supervised session and set goals with the participant for the upcoming week.

#### 2.7.2 Control group

During this initial 16-week period, control participants are provided a booklet with the prescribed stretching program to be completed 7 days per week to match the prescribed frequency of exercise sessions in the exercise group, e.g., 3 days/week supervised circuit training plus 4 days/week self-directed aerobic exercise. The stretching program is self-directed home-based with instruction provided at baseline testing only to orient them with the booklet. Similar to the exercise program, the stretches change every 4 weeks, where 4–5 stretches that target both upper and lower body are prescribed. Participants in the attention control group are offered the supervised component of the 16-week intervention following the initial 16-week period.

### 2.8 Intervention adherence

Exercise adherence is captured for the multicomponent exercise intervention by calculating (1) the percentage of supervised circuit trainings attended (out of 48 sessions) and self-directed exercise sessions completed (out of 64 sessions) and (2) relative dose intensities of supervised circuit trainings. Reasons for missed sessions will be documented throughout the study. However, to account for any unforeseen illness, family or medical emergency, or unplanned travel, all participants may have a total of 18 weeks to complete the 48 supervised exercise sessions. Participants are considered compliant if they attend ≥70% of the total prescribed number of supervised exercise sessions (e.g., attend ≥34 out of 48 sessions), ≥70% of the self-directed exercise sessions (e.g., complete ≥45 out of 64 sessions), and complete ≥70% of the supervised resistance sessions at the prescribed intensity. Participants are provided with monetary compensation for each optional muscle biopsy and testing visit and parking validation for every visit to Dana-Farber Cancer Institute.

### 2.9 Adverse events

Any expected and unexpected adverse events are or will be reported to the principal investigator, who then subsequently reports to the institutional review board of the Dana-Farber Cancer Institute as per policy.

### 2.10 Data monitoring and management

Data are monitored internally within the Dana-Farber Cancer Institute for timeliness of submission, completeness, and adherence to protocol requirements. Monitoring begins at the time of participant registration and will continue during protocol performance and completion. The study team collects, manages, and performs quality checks of the data. Potential audits or inspections may be conducted by the principal investigator or their designated representatives. All data are stored on a secure network drive using REDCap (Research Electronic Data Capture; Vanderbilt University), a Health Insurance Portability and Accountability Act-compliant web-based application hosted by Partners HealthCare Research Computing, Enterprise Research Infrastructure & Services, on password-protected computers. Any hard-copy data are stored in locked filing cabinets in card-access facilities. The results of this study will be presented in publication, conference, and invited speaker formats.

### 2.11 Sample size calculation

We will enrol a total of 80 eligible participants. Considering a 10% attrition rate, the sample size of the analysis population will be 72. We plan to accrue participants for 24 months. The Wilcoxon rank-sum test will be used to compare the score based on the frailty phenotype (37) between the two groups for the primary analysis. Table 4 shows the anticipated distribution of the score in each group (59). With a sample size of 72, the study will have 84% to detect the relative effect of 0.30 at a 0.05 two-sided alpha level. This power calculation was performed by the WMWssp package (60). For comparing each of the five criteria of the frailty score (binary outcomes), the sample size of 72 will provide 80% power to detect a moderate effect size (Cohen's *h* = 0.415) at a 0.05 two-sided alpha level (61). For each continuous outcome, two-sample *t*-tests will be used for the between-group comparison as the primary analysis. Log transformation may be considered, if appropriate. With a sample size of 72, the study will have 80% power to detect a moderate to large effect size (Cohen's *d* = 0.67) at a 0.05 two-sided alpha level.

**Table 4:** Sample size and power with the anticipated distribution of the frailty score (0–5).

Frailty status	Not frail (%)	Pre-frail (%)		Frail (%)			Total sample size	Relative effect	Power (%)
	0	1	2	3	4	5			
Attention control	15	63	17	4	1	0	72	0.30	84
Intervention	85	14	1	0	0	0			

### 2.12 Statistical analysis

This study will assess whether a 16-week, multicomponent exercise intervention will improve the frailty status of mPCa on ADT. Furthermore, we will assess the effect of the intervention on sarcopenia status, skeletal muscle-derived biomarkers, and cell proliferation rate.

A total of 80 eligible patients who complete the baseline assessment will be randomly assigned to the exercise or attention control groups in an allocation ratio of 1:1 using a permuted blocked design with varying block size (with investigators blinded to block size). The randomization will be stratified by frailty status (pre-frail vs. frail) at baseline assessment.

All analyses will be performed based on the intention-to-treat principle. We will repeat the same analyses with the subjects who complied with the protocol as secondary analyses. Baseline demographic and clinical measures, as well as baseline values of trial outcomes, will be presented by randomized intervention group; continuous measures will be summarized by mean (SD) or median (IQR) and categorical measures by frequencies (percent's). The randomized groups will be compared concerning baseline demographic and clinical variables using two-sample *t*-tests, Wilcoxon rank-sum tests, or Fisher's exact tests to confirm baseline comparability. These variables will be included in the adjusted analyses if clinically meaningful or statistically significant differences are identified.

The primary outcome is the score based on the Fried frailty phenotype (37), which consists of five criteria, and the score will take an integer value from 0 to 5. The Wilcoxon rank-sum test will be used as the primary analysis for comparing the score distribution between groups. Mean score and a corresponding 0.95 confidence interval (CI) will be calculated. We will also assess the intervention effect on each of the five criteria for the frailty phenotype for hypothesis generation purposes. For each, we will perform Fisher's exact test for the between-group comparison and estimate an odds ratio and a corresponding 0.95 CI. Regression analysis will be used to identify baseline factors associated with the outcomes, where proportional odds models will be used for the score as an ordered categorical variable with six categories, and logistic regression models will be used for each of the five criteria.

For other outcome continuous variables, mean, median, and corresponding 0.95 CIs will be calculated in each group. The primary comparison will be performed by two-sample *t*-tests. To assess the robustness of findings, Wilcoxon rank-sum tests will also be performed as secondary analyses. ANOVA models will also be used to identify baseline factors associated with the outcomes. Log transformation may be considered, if appropriate.

For longitudinal data, we will use generalized linear mixed-effect models with an appropriate link function (i.e., identity-link for continuous outcomes and logit-link for binary outcomes). The independent variables will include an indicator variable for the randomized intervention, time, and intervention-by-time interaction. Random intercepts (and random slopes) will be specified at the subject level. The within-subject correlation structure will be determined prior to evaluation of the between-group difference, using information criterion measures to determine the correlation structure providing the best model fit. In the model, the main effect of the intervention will test for group differences over the entire study period. Regarding missing values, the primary analysis will be all available data analysis, assuming that the missing mechanism is missing at random. As sensitivity analyses, we will apply multiple imputations (62).

## 3 Discussion

The FIERCE trial is the first exercise oncology study focusing on clinical and molecular analyses related to patients with mPCa during ADT treatment. The FIERCE trial employs a comprehensive analysis of the side effects of ADT treatment, assessing frailty and sarcopenia through questionnaires, physical evaluation, and imaging tests. In addition, muscle biopsies and plasma/serum will be used to evaluate biomarkers of the effects of exercise on frailty and sarcopenia. Potential modulating agents of physical exercise released by muscle cells will be examined on the LNCaP cell line, a human prostate cancer cell line that exhibits androgen-sensitive growth.

Men with mPCa undergoing treatment with ADT present with, among other side effects, loss of muscle mass, sarcopenia, and frailty, with a consequent increase in the risk of mortality (3, 4, 8). The reduction in muscle mass and frailty induced by ADT treatment has been an important clinical issue in patient management (63). In this regard, evidence supports the association of exercise-based interventions with global health improvements in patients undergoing ADT treatment with or without a diagnosis of metastases (10–12). Research has supported the feasibility and safety of physical exercise in men with metastatic or advanced prostate cancer (64–67). For example, in patients with advanced prostate cancer receiving ADT and patients with bone metastases, exercise training was able to increase muscle mass, strength, and aerobic capacity (67–70). Interestingly, Houben et al. (68) demonstrated that resistance exercise effectively countered ADT-induced side effects, regardless of protein supplementation combined with resistance exercise. Therefore, the beneficial effects of exercise among men with prostate cancer are mainly related to increased physical fitness (13) and reduced fatigue (71). Improvements in sexual and mental well-being (72, 73), as well as in quality of life (13, 74), have also been reported. However, some studies have shown no effect of exercise on sexual function (65) or quality of life (67, 75). Although these beneficial effects have been demonstrated across different training protocols and exercise types, multicomponent exercise-based training with a periodized prescription is promising for older adults and men with prostate cancer (69, 76).

Prior work has suggested that multicomponent exercise has significant potential to reduce ADT-induced side effects. In prostate cancer patients undergoing ADT, a combination of aerobic and resistance exercise enhances aerobic fitness and muscle strength, reduces fatigue, and preserves or increases lean mass, contributing to an increase in physical function and quality of life (69, 74, 77, 78). Furthermore, the addition of balance and flexibility exercises is particularly beneficial for older men with prostate cancer, to improve joint mobility, balance, and reduce the risk of falls (3). Above all, the maintenance or increase in muscle strength and mass, as well as the reduction in sarcopenia and frailty, is directly associated with a lower risk of mortality in men undergoing ADT treatment, regardless of cancer type (4, 7–9). Although multicomponent exercise may uniquely improve several components of health-related physical fitness in patients undergoing ADT treatment, the effect of multicomponent exercise on biochemical variables is poorly investigated. In this regard, Cormie et al. (77) observed a reduction in high-density lipoprotein (HDL), while Galvão et al. (69) observed a decrease in C-reactive protein, but in general, research has not identified changes in biomarkers of lipid and glucose metabolism (69, 77) or PSA concentrations (69, 74, 77, 78). Therefore, despite the beneficial effects of exercise training in men with prostate cancer, it remains unclear its impact on biochemical markers of metabolism or how exercise could modulate molecular pathways capable of mitigating the side effects of ADT treatment.

Potential molecular mechanisms that explain the beneficial effects of exercise on frailty and sarcopenia in men with mPCa are associated with low-grade inflammation (25). Conversely, changes in immunity favoring pro-inflammatory signaling are directly related to frailty (24). High levels of C-reactive protein have been consistently reported as biomarkers of frailty in older adults (79, 80). Corroborating, Navarro-Martínez et al. (5) showed that the concentrations of IL-6, C-reactive protein, and fibrinogen were positively associated with frailty in men with prostate cancer undergoing ADT. Interestingly, the authors reported that physical activity was also associated with frailty biomarkers. C-reactive protein and fibrinogen had higher serum concentrations in patients with moderate or light physical activity of <150 min per week (5). In addition, IL-6 was also higher in patients with lower walking speeds (5). Corroborating with frailty biomarkers, studies on the role of myokines in muscle tissue are still limited (28). Evidence has supported that myonectin and FGF21 were related to mitochondrial function and biogenesis (28, 81), SPARC, and brain-derived neurotrophic factor (BDNF) to muscle repair (82, 83), while increased muscle mass is associated with decorin, irisin, and FGF21 (84–86).

Moreover, the literature acknowledges the antitumor effects of exercise through myokines, particularly highlighting IL-6, IL-15, IL-10, SPARC, myostatin, irisin, decorin, and oncostatin M (87, 88). Kim et al. (89) demonstrated an increase in OSM but not SPARC and decorin after twelve weeks of exercise in prostate cancer patients treated with ADT. They used serum from these patients for *in vitro* testing, demonstrating that DU-145 prostate cancer cells had reduced proliferation when cultured with serum from trained patients (89). Similarly, using serum from prostate cancer patients who trained for 6 months reduced the proliferation of DU-145 cells. Myokine tests showed an increase in OSM and SPARC, while IGF-1 or IGFBP-3 remained unchanged after training (90). These antitumor effects have also been demonstrated using exogenous myokines. Investigation with PC-3 metastatic prostate cancer cells revealed that exogenous irisin reduced cell viability, while tumor growth and progression were reduced *in vivo*, suggesting that apoptotic pathways were enhanced by irisin (91). It was also demonstrated that exogenous decorin inhibited the growth of androgen-independent (PC3 and DU-145) and androgen-dependent (LNCaP) prostate cancer cells. In addition, the animal model treated with decorin showed delayed tumor development (92). However, research on the impact of myokines on prostate cancer is incipient, and the potential of myokines released from exercise with antitumor effects is still unclear. Furthermore, the molecular mechanisms by which exercise may contribute to the delay in muscle mass loss or gain related to ADT treatment or the impact of multicomponent exercise on cellular factors released by muscles still require further investigation and thus will be investigated in the FIERCE trial.

In this regard, the strengths of our study include the randomized controlled design and a comprehensive set of frailty and sarcopenia measures, including the utilization of muscle tissue biopsies to assess the impact of multicomponent exercise on molecular markers associated with sarcopenia. We also want to acknowledge that our study has several limitations. While the intention is to recruit frail and pre-frail participants as defined by the FRAIL questionnaire, this questionnaire is self-reported by the participant and may lead to misclassification. Although our intervention period allows us to determine changes in frailty and sarcopenia, this period would not permit us to determine if our intervention may be associated with reductions in comorbidities, metastatic modulation, or mortality. The partial clinic-based setting of the intervention may not be replicable in other environments, e.g., community programs and telehealth.

## 4 Conclusion

The FIERCE trial will evaluate the efficacy of a 16-week multicomponent exercise intervention encompassing supervised, clinic-based circuit training utilizing resistance exercises and self-directed aerobic exercise on frailty and sarcopenia, with an exploration of novel biomarkers at the intersection of frailty, sarcopenia, and disease progression. We hypothesize that multicomponent exercise will be effective in reducing the progression of frailty and sarcopenia, with an impact on molecular mechanisms related to the immune response and myokines, capable of improving patients' health and inhibiting prostate cancer cell development *in vitro*. Moreover, the exercise intervention includes multicomponent exercise periodization, which can enhance exercise prescription for men with mPCa by integration of various exercise modalities with structured progressions over time. The results of this study may corroborate the understanding of the antitumor effects of exercise on prostate cancer cells, in addition to the impact of periodization training to reduce sarcopenia and frailty in patients with mPCa, corroborating the development of future exercise guidelines for prostate cancer survivorship.
